# Intracardiac echocardiography–guided coronary sinus lead placement via left axillary vein access in a patient with persistent left superior vena cava and absent right superior vena cava

**DOI:** 10.1016/j.hrcr.2025.10.023

**Published:** 2025-10-22

**Authors:** Arooj Khan, Mandeep Bhargava, Bryan Baranowski, Shady Nakhla

**Affiliations:** Department of Cardiovascular Medicine, Section of Electrophysiology, Heart, Vascular, and Thoracic Institute, Cleveland Clinic, Cleveland, Ohio

**Keywords:** Persistent left superior vena cava, Coronary sinus lead, Intracardiac echocardiography, Cardiac resynchronization therapy, Contrast-sparing approach

**Key Teaching Points**

• Persistent left superior vena cava (PLSVC) with absent right superior vena cava is a rare congenital anomaly that poses significant challenges for coronary sinus (CS) lead placement during cardiac resynchronization therapy.
• Intracardiac echocardiography (ICE) introduced via axillary vein access can provide high-resolution, real-time visualization of the CS and its branches, facilitating safe and effective lead delivery in complex venous anatomies.
• The use of ICE minimizes the need for iodinated contrast, which is particularly advantageous in patients with chronic kidney disease or contrast allergy. In this case, only 10 mL of contrast was required for selective branch venography.
• Active-fixation left ventricular leads and adjunctive tools (eg, extended hook sheath with inner subselecting catheter) may enhance stability and successful lead placement in patients with anomalous venous return such as PLSVC.

## Introduction

Persistent left superior vena cava (PLSVC) is the most common thoracic venous anomaly, present in approximately 0.3%–0.5% of the general population and up to 4.4% of patients with congenital heart disease.^1^ The absence of a right-sided superior vena cava (SVC) in combination with PLSVC is rare.^2^ This anatomic variation presents a technical challenge for coronary sinus (CS) lead placement during cardiac resynchronization therapy (CRT) device implantation. We present a case where intracardiac echocardiography (ICE) was used via left axillary vein access to facilitate CS lead placement, minimizing the use of contrast in a patient with chronic kidney disease and absent right SVC.

## Case report

A 78-year-old male with a medical history of coronary artery disease, previous coronary artery bypass grafting, multiple percutaneous coronary interventions, ischemic cardiomyopathy (left ventricular [LV] ejection fraction 15%), left bundle branch block, persistent atrial flutter, hypertension, and stage 3 chronic kidney disease was referred for CRT with a defibrillator implantation.

**Figure 1:**
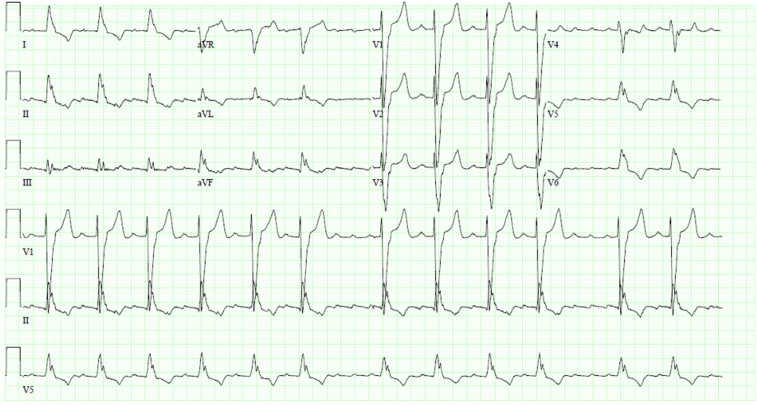
Baseline 12-lead electrocardiogram showing atrial flutter with variable conduction and a typical left bundle branch block.

Baseline electrocardiogram (Figure 1) showed atrial flutter with variable conduction and typical left bundle branch block. He had recent hospitalizations for decompensated heart failure, and CRT with a defibrillator implantation was recommended.

**Figure 2:**
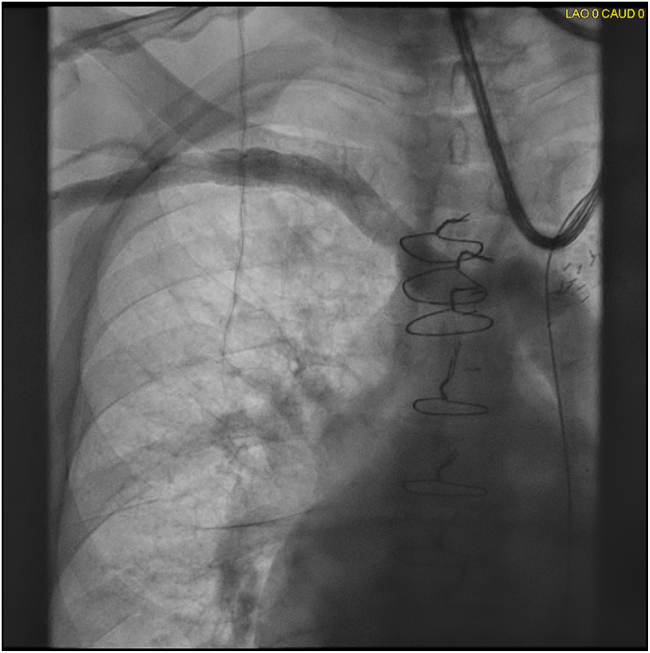
Right upper extremity venogram demonstrating a bridging vein from the right subclavian vein to the persistent left superior vena cava and absence of a right-sided superior vena cava.

Review of a previous chest computed tomography from 2015 revealed a PLSVC. In the electrophysiology laboratory, a right upper extremity venogram demonstrated the absence of a right-sided SVC and drainage of the right subclavian vein into the LSVC via a bridging vein (Figure 2).

**Figure 3:**
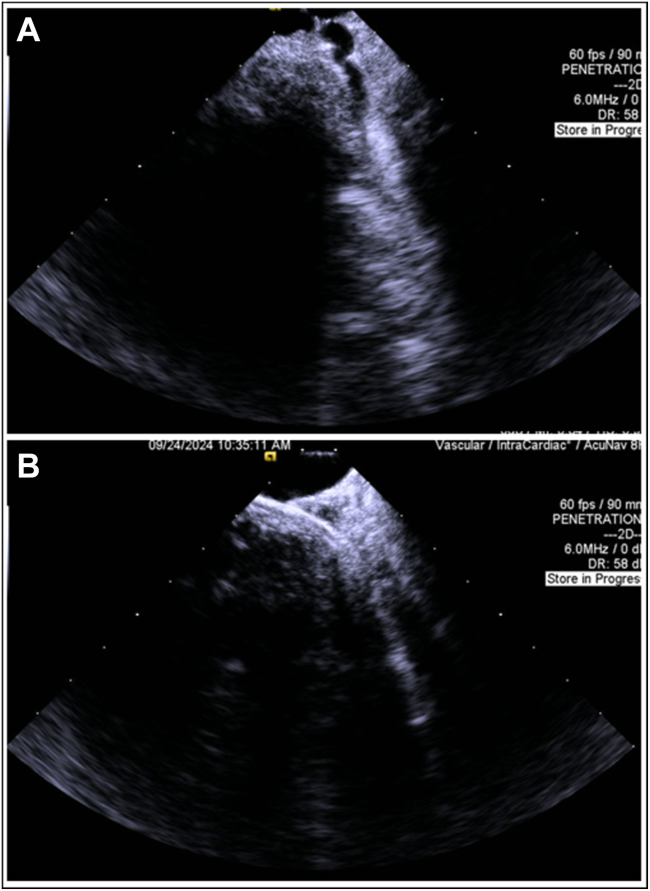
**A–B:** Intracardiac echocardiography visualization of the coronary sinus (CS) anatomy and successful cannulation of a lateral CS branch using an extended hook sheath.

Given the patient’s renal function, we opted for a contrast-sparing approach using ICE to guide CS cannulation and LV lead placement. After placing the right ventricular implantable cardioverter-defibrillator lead in the apex, an 8F ICE catheter was introduced via one of the left axillary vein accesses. A long sheath was advanced to minimize deflection and facilitate the smooth passage of the ICE catheter into the LSVC and subsequently into the CS. ICE imaging identified the middle cardiac vein and a lateral branch at the 3–4-o’clock position (Figure 3). Although color Doppler was applied, high-resolution 2-dimensional ICE imaging provided excellent anatomic detail and was primarily relied upon for branch identification.

Owing to the small caliber of the middle cardiac vein, the lateral branch was selected for LV lead deployment. A total of 10 mL of iohexol (Omnipaque) contrast was used for selective branch venography, although carbon dioxide may be considered an alternative in similar cases.

**Figure 4:**
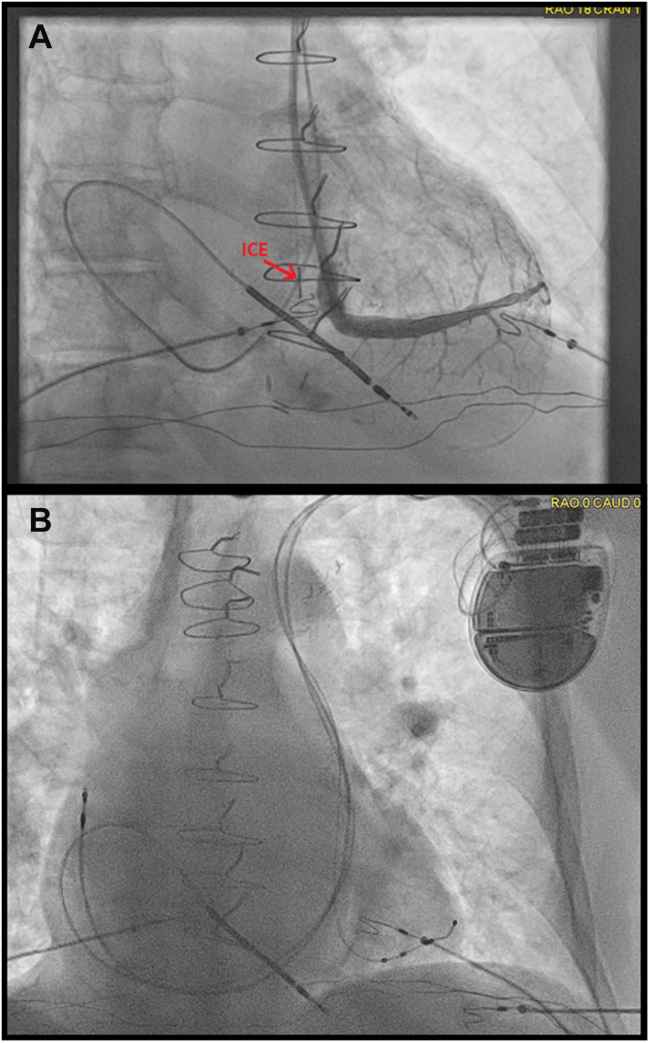
**A–B:** Intracardiac echocardiography catheter positioned within the coronary sinus body during selective venography of a lateral branch and final fluoroscopic imaging demonstrating optimal lead placement.

**Figure 5:**
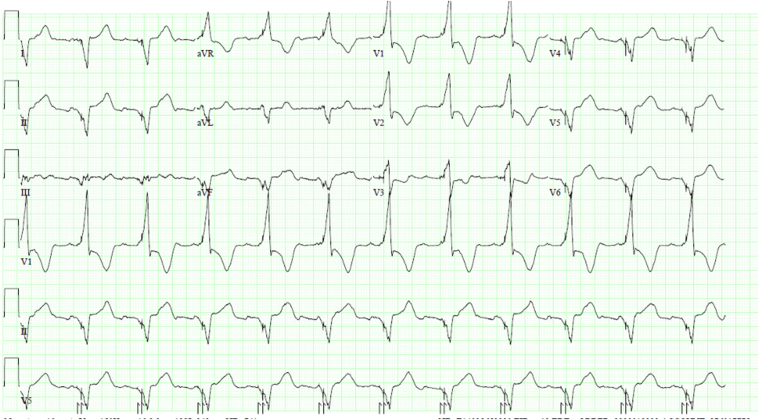
Postimplant electrocardiogram demonstrating effective biventricular pacing.

Using real-time ICE and fluoroscopy, the lateral branch was successfully cannulated with an extended hook sheath, in combination with a 90° inner subselecting catheter. The LV lead was positioned with satisfactory thresholds and stability (Figure 4). A Medtronic 4798 active-fixation lead was selected to maximize stability given the anomalous venous anatomy. Final LV pacing threshold was (0.5 V at 0.5 ms), with stable impedance (430 Ω). The paced QRS morphology showed appropriate biventricular capture (Figure 5).

## Discussion

This case highlights a novel and practical approach for CRT lead implantation in a patient with challenging venous anatomy. PLSVC with an absent right SVC significantly alters standard access routes for CS cannulation. Conventional use of contrast venography poses risks in patients with renal impairment, necessitating alternative imaging techniques.^2^

ICE provides high-resolution, real-time visualization of cardiac structures and venous anatomy and can be advanced via peripheral venous access.^3^ In this case, ICE enabled the visualization of the CS and its branches from within, allowing precise localization and successful lead placement without contrast administration.

Previous case reports have described the use of ICE in guiding CS cannulation,^3^, ^4^, ^5^ but few have reported its use through an axillary approach in patients with a PLSVC and absent right SVC to specifically identify the os of the coronary vein for lead placement. Importantly, conduction system pacing was felt unfeasible owing to the lack of right-sided SVC and the lack of a delivery catheter long enough and with enough turns to facilitate implant of such a lead through an LSVC. An alternative pacing strategy that may be feasible in this case would be a leadless LV lead (WiSE-CRT) but was not pursued.^6^

This method demonstrates feasibility and safety and may be especially advantageous in patients with renal dysfunction or contrast allergy.

## Conclusion

ICE can serve as a valuable adjunct in complex CRT device implantation scenarios, particularly in patients with anomalous venous anatomy and contraindications to iodinated contrast. In this case, the ICE-guided approach via axillary access enabled successful and safe LV lead placement in a patient with a PLSVC and no right-sided SVC, avoiding contrast-related nephrotoxicity.^3^, ^4^, ^5^

## Disclosures

The authors have no conflicts of interest to disclose.
